# Supplementary material for: Barriers and facilitators to utilizing HIV prevention and treatment services among migrant youth globally: A scoping review
Source: PLOS Glob Public Health. 2024 Feb 14;4(2):e0002851. doi: 10.1371/journal.pgph.0002851 (PMC10866458; doi:10.1371/journal.pgph.0002851)
Supplement: S1 Text — (DOCX) [file pgph.0002851.s002.docx]

**S1 Text. Search term keywords**

PubMed:

1. "Transients and Migrants"[Mesh] OR "Refugees"[Mesh] OR "Emigrants and Immigrants"[Mesh] OR "Emigration and Immigration"[Mesh] OR “Migration*”[tw] OR "Immigrants*"[tw] OR "Transients*"[tw] OR "Refugees*"[tw] OR "Asylum Seekers*" OR “Emigrants”[tw] OR “Human Migration”[tw] OR “Seasonal worker”[tw] OR “residential mobility”[tw] OR “non-permanent migration”[tw] OR “mixed movement”[tw] OR “mixed flows”[tw] OR “composite movement”[tw]
2. AND “Adolescent”[MeSH] OR “Adolescent”[All Fields] AND “Young”[All Fields] AND “Persons”[MeSH] OR “Persons”[All Fields] OR “People”[All Fields] OR “Adolescent”[MeSH] OR “Adolescent”[All Fields] OR “Teenager”[All Fields] OR “Adolescent”[MeSH] OR “Adolescent”[All Fields] OR “Youth”[All Fields]
3. AND “HIV”[MeSH] OR “HIV”[All Fields] OR HIV[tiab] OR "HIV"[tw] OR “human”[All Fields] AND “immunodeficiency”[All Fields] AND “virus”[All Fields] OR “human immunodeficiency virus”[All Fields] OR “acquired immunodeficiency syndrome”[MeSH] OR “acquired”[All Fields] AND “immunodeficiency”[All Fields] AND “syndrome”[All Fields] OR “acquired immunodeficiency syndrome”[All Fields] OR “hiv aids”[All Fields]

Web of Science

1. ( ( ( ( ( ( ( ( ( ( ( ( ( ( (ALL= (transients)) OR ALL= (migrants)) OR ALL= (refugees)) OR ALL= (emigrants)) OR ALL= (immigrants)) OR ALL= (emigration)) OR ALL= (immigration)) OR ALL= (migration)) OR ALL= (asylum seekers)) OR ALL= (human migration)) OR ALL= (seasonal worker)) OR ALL= (residential mobility)) OR ALL= (non permanent migration)) OR ALL= (mixed movement)) OR ALL= (mixed flows)) OR ALL= (composite movement)
2. AND ( ( ( ( (ALL= (adolescent)) OR ALL= (young person)) OR ALL= (young people)) OR ALL= (teenager)) OR ALL= (teen)) OR ALL= (youth)
3. AND ( ( ( ( (ALL= (hiv)) OR ALL= (immunodeficiency virus)) OR ALL= (human immunodeficiency virus)) OR ALL= (acquired immunodeficiency syndrome)) OR ALL= (hiv aids))

Filters: English Language, Publication Year (2012-current)
