# Supplementary material for: Barriers and facilitators to utilizing HIV prevention and treatment services among migrant youth globally: A scoping review
Source: PLOS Glob Public Health. 2024 Feb 14;4(2):e0002851. doi: 10.1371/journal.pgph.0002851 (PMC10866458; doi:10.1371/journal.pgph.0002851)
Supplement: S1 Table — (DOCX) [file pgph.0002851.s003.docx]

# **S1 Table. Data extraction template**

| **Background information** |
| --- |
| Study ID:  Title:  Country in which the study was conducted:  Notes: |
| **Methods** |
| Category: (Qualitative/Quantitative/Mixed-Method/Multi-Method)  Study aim:  Study design:  Data collection methods:  Data analysis techniques: |
| **Participants** |
| Sample size:  Adolescent and/or youth population ≥70% of study population?: (Yes/No)  Migrant sub-population: (Refugee/Immigrant/International Student/Migrant Worker/Other)  Population description: |
| **Results** |
| Key findings:  Barriers:  Facilitators:  Other key information:  Notes: |
