# Supplementary material for: Barriers and facilitators to utilizing HIV prevention and treatment services among migrant youth globally: A scoping review
Source: PLOS Glob Public Health. 2024 Feb 14;4(2):e0002851. doi: 10.1371/journal.pgph.0002851 (PMC10866458; doi:10.1371/journal.pgph.0002851)
Supplement: Attachment — Submitted filename: Response to Reviewers.pdf [file pgph.0002851.s005.pdf]

December 8, 2023

Dear Dr. Escandón and PLOS Global Health Editorial Team,

On behalf of my co-authors, Kevin Li, Natasha Thaweesee, Allison Kimmel, Emily Dorward, I would like to thank PLOS Global Health for the thoughtful feedback on our manuscript. In response to the comments received, we are submitting a revised version of our manuscript for consideration.

As recommended, we have listed below the reviewers' comments (in ***italicized black***) and included our point-by-point responses to each (in **red**). Separately, we have uploaded a marked-up copy of the manuscript that highlights changes made to the original version, titled 'Revised Manuscript with Track Changes', an unmarked version of the revised paper without tracked changes, titled 'Manuscript', and a revised Figure 2.

We have no conflicts of interest, and verify that this manuscript has not been submitted elsewhere. Thank you for your continued consideration of this manuscript.

Sincerely,

Anita Dam

Anita Dam, MSPH
Technical Advisor, Introduction & Access of HIV Prevention Technologies
USAID's Office of HIV/AIDS, Research Division
andam@usaid.gov
+1 (571) 218-2601

***Please note that all line numbers referenced in our responses correspond to the document titled "Revised Manuscript with Track Changes", not the document titled "Manuscript"**

Reviewer #1:

The topic of the study is relevant, the intersection of young people and immigrants is a global public health problem that needs to be tackled to mitigate the HIV epidemic. The manuscript is well written and its methodology is sound.

Major comment:

Search strategy - Although I understand the difficulties of reading articles in languages other than English, given that most massive immigration events take place in non-English-speaking countries ("global South"), I think that the decision of excluding articles in other languages in the Search ("language filter") imposes an important limitation on the work. The authors could have carried out the search without excluding articles on the basis of language and excluding them in a second step. By doing that, readers could at least have an idea of the amount of information/articles that were not evaluated on the basis of language. This strategy would also be better suited to a global perspective and would show the intention to be more inclusive of other countries and communities.

- Response: We sincerely thank you for your feedback. We did not employ a language filter when carrying out our searches on the two databases. Thirteen articles were included that were not written in English, which we excluded during the full text review process, as outlined in our PRISMA flowchart. To better acknowledge a global perspective, we expanded upon this limitation in our Discussion (see lines 641 to 642).

Minor comments:

Methods: Add reference to Covidence software.

- Response: Thank you for this comment. We have added a reference to Covidence (See line 196)

Results – Figure 2 – Suggest adding sample sizes (n) in the boxes indicating the number of studies reporting each Variable

- Response: Thank you for this comment. As suggested, we have added the sample sizes indicating the number of studies in Figure 2 (see revised Figure 2).

Limitation paragraph (lines 501-503): "Thirteen publications were excluded at the full-text review stage for not being written in English, but 85% of included publications (n=17) were published from a study location that does not use English as an official language." I was confused, the language was not used as a filter in the Search? If yes, those 13 publications had "escaped" the filter? Moreover, it is hard to conclude anything meaningful based on those on the statement that 85% of the publications included were from non-English-speaking countries.... the real question is how many publications were not included due to the language restriction. Even more, although the purpose and the methods of this study are sound, it would be valid to raise some discussion on how we do not have data on locations where forced and massive immigration is happening.

- Response: We sincerely thank you for your input. We acknowledge this concern and limitation in our paper. As mentioned in our response to the prior comment, 13 publications were not included in our review due to language restrictions. In concordance with your suggestion, we elaborate on this exclusion as a limitation to our review (see lines 640 to 642). We have also removed the aforementioned lines, as we agree that it is difficult to conclude anything meaningful from the statement.

Reviewer #2:

Did the authors collaborate with a public health or medical librarian to formulate the search terms?

- Response: We sincerely thank you for this comment. We collaborated with Teresa Yeh, faculty at the Johns Hopkins Bloomberg School of Public Health and expert in systematic reviews, to formulate our search terms. To reflect this contribution, we have included this detail in our acknowledgments (see line 668).

What was the rationale behind excluding articles published before 2021?

- Response: Thank you for this comment. To clarify, we chose to include articles published between 2012-2022, not 2021-2022 (see line 187). We chose to exclude articles published before 2012 to ensure that the papers we were reviewing were not outdated in terms of available health services or sociopolitical context. As such, we chose 2012 as our cut-off year to include peer-reviewed literature published within the past 10 years.

When was the search strategy put into action?

- Response: Thank you for considering this, and bringing this comment to our attention. Our search strategy was put into action on October 19, 2022. For additional clarity, we have added this date to our Methods section (see line 186)

Is there a specific reason for utilizing only two databases, which might have potentially resulted in overlooking significant publications?

- Response: Thank you for this comment. We chose to utilize only two databases due to a lack of capacity to review a larger amount of papers. In collaboration with Teresa Yeh, an expert in systematic reviews, we decided that the breadth of these two databases would sufficiently represent this topic. We have included a statement on this limitation in our discussions section (see lines 638 to 639)

For studies where the participant age range extended beyond youth, how was the 70% threshold determined for the 15-24 age group?

- Response: Thank you for this comment. We chose 70% as a threshold after reviewing other scoping reviews focused on youth populations, which chose thresholds at similar percentages. Examples include a scoping review from Green et al., which considered a study population to be "focused on youth aged 15–24" if "at least two-thirds of the sample" were between 15-24 years old, and Baigry et al. in PLOS Global Health, which included studies if >80% of the study population were between the ages of 15-24.

The inclusion criterion, "Discusses HIV prevention and treatment services," lacks context. The same ambiguity applies to the subsequent inclusion criterion, "Discusses barriers or facilitators to HIV prevention and treatment services."

- Response: Thank you for this comment. Due to the exploratory nature of our topic, we intended for this to be a scoping review to be inclusive in capturing the available data. As such we chose this language for our inclusion criteria in order to include a wider range of studies.

Reviewer #3:

Line 93: "15 to 24"

- Response: Thank you for this correction. We have corrected this error in Line 93.

Line 96: "across?"

- Response: Thank you for this correction. We have corrected this error in Line 96.

Line 128: "barriers, to and facilitators of, accessing"

- Response: Thank you for this correction. We have corrected this error in Line 137, and in subsequent lines where this language appears (see line 139).

Line 135: "What is the positionality of the researchers relative to the topic?"

- Response: Thank you for this question. We were hoping you could clarify if this was in relation to the authors' affiliations, or the determination to conduct a scoping review versus a systematic review.

Line 139: "15 to 24 years"

- Response: Thank you for this correction. We have corrected this wording in line 157.

Line 142: "Long reference. I would rewrite to avoid this long strange reference in the text."

- Response: Thank you for this correction. We have rewritten this sentence accordingly in line 160.

Line 153: "Why this timeframe?"

- Response: Thank you for this question. We chose to exclude articles published before 2012 to ensure that the papers we were reviewing were not outdated in terms of available health services or sociopolitical context. As such, we chose 2012 as our cut-off year to include peer-reviewed literature published within the past 10 years.

Line 155: "Throughout the manuscript you need to make sure you include the units, i.e. here the units are "years". So this should be written as "15-24 years". However in a sentence, as below (see my highlight) it should be written as '15 to 24 years'"

- Response: Thank you for this correction. We have corrected this error in line 173, and in other lines where this language appears (see lines 128 and 178).

Line 160: "Rationale for this proportion? Are there previous prominent researchers/papers that has used this rationale?"

- Response: Thank you for this comment. We chose 70% as a threshold after reviewing other scoping reviews focused on youth populations, which chose thresholds at similar percentages. Examples include a scoping review from Green et al., which considered a study population to be "focused on youth aged 15–24" if "at least two-thirds of the sample" were between 15-24 years old, and Baigry et al. in PLOS Global Health, which included studies if >80% of the study population were between the ages of 15-24.

Line 163: "Why only two databases? This is limiting and unusual."

- Response: Thank you for this comment. We chose to utilize only two databases due to a lack of capacity to review a larger amount of papers. In collaboration with Teresa Yeh, an expert in systematic reviews, we decided to search the two most inclusive databases without being duplicative. We have included a statement on this limitation in our discussions section (see lines 638 to 639)

Line 164: "Did the researchers have input from a researcher librarian or expert?"

- Response: We sincerely thank you for this comment. We collaborated with Teresa Yeh, faculty at the Johns Hopkins Bloomberg School of Public Health and expert in systematic reviews, to formulate our search terms. To reflect this contribution, we have included this detail in our acknowledgments (see line 668).

Line 172: "It is very good at removing duplicates, but rarely removes ALL duplicates. Is this actually true?"

- Response: Thank you for this concern. During the title and abstract screening process, we also ensured all duplicates were removed. We have updated our methods to reflect this (see line 196)

Line 179: "Who was this?"

- Response: Thank you for this question. We have updated our methods section to include the full name of the reviewer, Anita Dam, in line 200.

Line 180: "Who was this?"

- Response: Thank you for this question. We have updated our methods section to include the full name of the reviewer, Allison Kimmel, in line 200.

Line 183: "How was the data managed? In what platform or software was it synthesized, stored, developed into themes? The researchers would benefit from following a reporting checklist to ensure all the required content is reported in this review."

- Response: Thank you for this question. Data was managed, synthesized, stored, and developed into themes using Microsoft Excel and matrix analysis. We have included a sentence in our methods section to clarify this (see lines 212 to 213).

Lines 191-192: "Can the authors comment on how this approach is a limitation, particularly given the scoping review approach?"

- Response: Thank you for this question. The authorship team agreed that since the review was exploratory in nature, it would be fitting to employ qualitative coding. The two-step process outlined in lines 202-204 allowed us to analyze our data without pre-existing notions while also employing codes that allow for the effective use of the Social-Ecological Model as an analytical framework. As other scoping reviews covering similar topics have used this form of analysis, like this scoping review from Tirado et al., we felt justified in our choice of analytical method.

Line 199: "It is poor grammar to commence sentences with a numeral. There are multiple instances of this throughout the manuscript. Please rewrite to avoid this throughout the manuscript."

- Response: Thank you for this correction. We have corrected this error in line 226, and in subsequent instances where this error appears (see line 227).

Line 200: "Do not start sentences with numbers."

- Response: Thank you for this correction. We have corrected this error in line 227.

Lines 202-203: "Repetitive of Figure 1. Doesn't need to be written in such detail here."; "Also, the numbers do not match what is presented in Figure 1."

- Response: Thank you for this comment. We have removed this sentence for concision.

Line 210: "used data from"

- Response: Thank you for this correction. We have corrected this error in lines 244 to 245.

Line 218: "Write numbers under 10 out as words, i.e. 6 should be written as 'six'. This occurs several times throughout the manuscript and needs addressing at each point."

- Response: Thank you for this correction. We have corrected this error in line 234, and in subsequent lines where this type of error appears.

Line 234: "six"

- Response: Thank you for this correction. We have corrected this error in line 273.

Line 243: "Every factors can either facilitate or prevent engagement. I think the authors would be better reframing the results as 'factors which influence' given each factor can be positive or negative depending on the circumstances."

- Response: Thank you for the suggested edit. We have rephrased our results section accordingly to use “factors which influence ____” (see lines 301, 318, 344, 367 to 368, 381 to 382, 398, and 412).

Line 244: “I think this is the wrong word/grammar choice for this sentence and needs revision to clarify the meaning.”

- Response: Thank you for this correction. The sentence has been revised accordingly (See line 303).

Line 248-249: “Am I correct in understanding rural migrants are people who have moved within a country? However, could the authors please clarify how immigrants and migrant workers are different classifications?”

- Response: Thank you for your comment. Yes, your understanding of the definition of rural migrant is correct. For your second question on clarifying classifications, immigrants move from one country to another, whereas migrant workers move both domestically and internationally for the express purpose of work; we chose to use the terminology for migrant subpopulations employed by the 20 included publications.

Line 265: “What does this mean? It is non-standard terminology which needs more explanation for the reader. Is this a concept the participants put forward, or the authors interpretation of the content?”

- Response: Thank you for this question. “Soon-to-be-successful” was wording used by Bernays et al. (35) to describe the ambitious identities adopted by the youth in this study. We have rewritten this sentence for clarification (see line 336).

Line 268: “Who defines it as hostile? Hostile in what way?”

- Response: Thank you for pointing out the ambiguity of our word choice. We have replaced “hostile” with “challenging”, which Bernays et al. uses to describe the conditions that their study participants face (see line 339).

Line 269: “Mechanism for coping within what context? What are they coping with?”

- Response: Thank you for pointing out the ambiguity of our word choice. We have rewritten this sentence accordingly (see line 340).

Line 274: “As previously indicated, this wording might be better replaced with "influencing access to HIV services"”

- Response: Thank you for the suggested edit. We have rephrased our results section accordingly to use "factors which influence ____" (see lines 301, 318, 344, 367 to 368, 381 to 382, 398, and 412).

Lines 276-277: "Education and employment status are closely related. Can the authors comments on whether these papers also reported education and whether these papers identified this link for their populations?"

- Response: Thank you for this comment, we acknowledge the link between education and employment status. While levels of educational attainment were measured among participants in all four publications (41, 42, 46, 49), none explicitly discussed the relation between the two socioeconomic factors.

Line 287: "The authors state there are three studies supporting this theme, however, then go on to state below that the data all came from the same study. So in fact, there is only one study supporting this theme, but reported across three papers? The way this is worded here is misleading and needs to be revised."

- Response: Thank you for the suggested edit. We have revised this sentence accordingly for clarity (see line 368).

Line 310: "It seems in general there are several themes where 'Community' or 'Relationships' etc are discussed but where there is not a convergence of the data on a particular (positive/negative) way the factors influences the behavior. Let me explain further, for example, three studies mention religion, but they all mention different aspects of how religion influences behavior or dialogue about HIV and prevention. The data don't show a common theme, but just that religion (in some way) is important. This makes it hard to use the findings to support a strategy/intervention design because there isn't a clear conclusion of what the underlying issue is. Can the authors comment on this? Do the authors confirm the data supports the findings they have derived?"

- Response: Thank you for this consideration and question. We acknowledge that there is not a convergence of data in a particular way for certain themes within the Community or Relationships level. Our goal with conducting a scoping review was to explore what is available in the literature about this topic. As such, we do not expect intervention design to be based on our research. Any strategy or intervention should be context specific.

Lines 335-336: "Further to my previous comment, here is an example of where two studies report the same issue, which means public health practitioners have an actionable intervention target. This level of theme/subtheme development is not evident across all the themes."

- Response: Thank you for providing an example for your previous comment. We have addressed this in our response above.
